# Acknowledgement of manuscript reviewers

**DOI:** 10.1186/1743-7075-11-7

**Published:** 2014-03-12

**Authors:** Lucy Abel, Ahmed Bakillah, M Mahmood Hussain

**Affiliations:** 1 BioMed Central, 236 Gray’s Inn Road, WC1X 8HB London, UK; 2 Department of Cell Biology, SUNY Downstate Medical Center, 450 Clarkson Ave, 11203 Brooklyn, NY, USA

## Abstract

**Contributing reviewers:**

We and the Editorial Board acknowledge and thank all reviewers for their active participation and contribution during 2013. We greatly appreciate their dedication and behind the scenes contribution. It is largely due to their support and expertise that we have been able to publish high-standard manuscripts. We would also like to thank authors for choosing Nutrition & Metabolism and contributing their cherished work.

## 

Niyazi Acar

France

Marcia Aguila

Brazil

Saeed Akhtar

Kuwait

Nayef Algharaibeh

Jordan

David Allison

United States of America

Emma Allott

United States of America

Futwan Al-Mohanna

Saudi Arabia

John Aloia

United States of America

Ezequiel Alvarez

Spain

Anton Amann

Austria

Theodore Angelopoulos

United States of America

Jose Antonio

United States of America

Alex Aregbesola

Finland

Vasilios Athyros

Greece

Sarah Auharek

Brazil

Salman Azhar

United States of America

Thomas Badger

United States of America

Jasmohan Bajaj

United States of America

Ahmed Bakillah

United States of America

Michiel Balvers

Netherlands

Tyler Barker

United States of America

Neal Barnard

United States of America

Masood Bazrgar

Iran

Elizabeth Bertone-Johnson

United States of America

H Tanju Besler

Turkey

Sheng Bi

United States of America

Xin Bi

United States of America

William Blaner

United States of America

Kathryn Bradbury

United Kingdom

George Bray

United States of America

Christopher Brey

United States of America

Tom Broderick

United States of America

Ruud Buijs

Mexico

Thomas Burkey

United States of America

L. C. Cameron

Brazil

Luciana Caperuto

Brazil

Lucile Capuron

France

Axel Carlsson

Sweden

Christian Carpéné

France

Sue-Joan Chang

Taiwan

Ubon Cha'on

Thailand

Oliver Chen

United States of America

Juei-Tang Cheng

Taiwan

Myung-Sook Choi

Korea South

Michael Chua

Philippines

Streamson Chua

United States of America

Katherine Cianflone

Canada

Graeme Close

United Kingdom

Kate Collison

Saudi Arabia

Paola Antonia Corsetto

Italy

Paola Costelli

Italy

Philippe Costet

United States of America

Sherry Dadsetan

Denmark

Kezhi Dai

United States of America

GM Dallinga-Thie

Netherlands

Undurti Das

United States of America

Nicholas Davidson

United States of America

Brenda Davy

United States of America

Carlos De Miguel

Spain

Nicole de Wit

Netherlands

Tamás Decsi

Hungary

Yvonne Denkins

United States of America

Wim Derave

Belgium

pskevi Detopoulou

Greece

Patrick D'Haese

Belgium

Marco Fabrício Dias Peixoto

Brazil

Jared Dickinson

United States of America

Ryan Dilger

United States of America

Hans Dreyer

United States of America

Alan Ducatman

United States of America

Klaus Eder

Germany

Abdalla El-Mowafy

Egypt

Munechika Enjoji

Japan

Mara Epstein

United States of America

Ramon Estruch

Spain

Peter Eu

Australia

Chris Exley

United Kingdom

Chris Fahs

United States of America

Germana Falcone

Italy

Longhou Fang

United States of America

Anthony Fardet

France

Richard Feinman

United States of America

Milagro Fermin

Spain

Victoria Foletta

Australia

Benedicte Fontaine-Bisson

Canada

Flavio Francini

Argentina

Zhengwei Fu

China

Gotthold Gäbel

Germany

Nicholas Gabler

United States of America

Alessandra Gambero

Brazil

Mary C. Gannon

United States of America

Javier Gisbert

Spain

Julia Goedecke

South Africa

Edward Gorham

United States of America

Louise Grahnemo

Sweden

Aldo Grefhorst

Netherlands

Sudhiranjan Gupta

United States of America

Robert Hackman

United States of America

Tiruneh Hailemariam

United States of America

Kei Hamazaki

Japan

Robert Harris

United States of America

Earl Harrison

United States of America

Peter Havel

United States of America

Alan Hayes

Australia

Aibin He

United States of America

Helen Hermana Hermsdorff

Brazil

Ernesto Hernandez

United States of America

Marvin Heuer

United States of America

Michael Holick

United States of America

Jane Hoover-Plow

United States of America

Sander Michel Houten

United States of America

Adela Hruby

United States of America

Yong Hu

United States of America

M. Mahmood Hussain

United States of America

Fotios Iliadis

Greece

Hiroyasu Inoue

Japan

Jahangir Iqbal

United States of America

Helene Jacques

Canada

Sushil Jain

United States of America

Yvonne Jeanes

United Kingdom

Eui-Bae Jeung

Korea South

Bo Ji

United States of America

Xian-Cheng Jiang

United States of America

Ravin Jugdaohsingh

United Kingdom

Pierre Julien

Canada

Afshan Kaleem

Pakistan

Douglas Kalman

United States of America

Nishan Kalupahana

Sri Lanka

Alvin Kamili

Australia

Hideaki Kaneto

Japan

Tilakavati Karupaiah

Malaysia

Hisnaori Kato

Japan

Hisayoshi Kawahara

Japan

Susanne Keiding

Denmark

Roya Kelishadi

Iran

Darshan Kelley

United States of America

Sander Kersten

Netherlands

Ulkan Kilic

Turkey

Jung Han Kim

United States of America

KyoungKon Kim

Korea South

Mi-Hyun Kim

Korea South

Sofia Klingberg

Sweden

Richard Kollmar

United States of America

Suminori Kono

Japan

Hannu Koponen

Finland

Mark Kotowicz

Australia

Sandra Kraljevic Pavelic

Croatia

Richard Kreider

United States of America

Natraj Krishnan

United States of America

Alan Kristal

United States of America

Herculina Salome Kruger

South Africa

Takashi Kudo

United States of America

Anil Kulkarni

United States of America

Chao-Qiang Lai

United States of America

Wouter H. Lamers

Netherlands

Andrew Lane

United States of America

Stuart Lanham

United Kingdom

Martine Laville

France

Donald Layman

United States of America

Mi-Kyung Lee

Korea South

Margret Leosdottir

Sweden

Tung Ming Leung

United States of America

Duo Li

China

Bernt Lindahl

Sweden

Fabio Lira

Brazil

George Liu

China

Jing-Chen Liu

China

Yuefei Liu

Germany

Eulogio J. Llorent-Martínez

Spain

Dorothea Loesel

Germany

Yan-Ru Lou

Finland

Thomas Lutz

Switzerland

David Ma

Canada

Denise Mafra

Brazil

Sameh Magdeldin

Japan

Anssi Manninen

Finland

Rachel Marion-Letellier

France

Brian Martin

Canada

Steve McAnulty

United States of America

Gethin McBean

Ireland

Ann McGinty

United Kingdom

Robin McGregor

New Zealand

Roger McLeod

Canada

Ronald Mensink

Netherlands

Antti A Mero

Finland

Joris Michiels

Belgium

Catherine Milte

Australia

Bettina Mittendorfer

United States of America

Carolyn Moore

United States of America

Lisa Moran

Australia

Anselmo Moriscot

Brazil

Naima Moustaid-Moussa

United States of America

Josephia Muindi

United States of America

David Muller

France

Isao Muraki

United States of America

Mariko Murata

Japan

Andrew James Murray

United Kingdom

Kamatham Akhilender Naidu

India

Harikumaran Nair Nair

India

Alireza Nakhaee

Iran

Forrest Nielsen

United States of America

Jens Niklas

Germany

Michalis Nikolaidis

Greece

Itzhak Nissim

United States of America

Luciana Nobre

Brazil

Estela Maria Novak

Brazil

Everson Nunes

Brazil

Haruhiko Osawa

Japan

Tone-Kari Østbye

Norway

Henrik Oster

Germany

Noriyuki Ouchi

Japan

Mototsugu Oya

Japan

Xiaoyue Pan

United States of America

Athanasios Papadopoulos

Greece

Stefan Pasiakos

United States of America

Dominik Pesta

United States of America

Andreas Peter

Germany

Stuart Phillips

Canada

Stephen Phinney

United States of America

Olivia Phung

United States of America

Gustavo Duarte Pimentel

Brazil

W Pogozelski

United States of America

Rena Pollack

United States of America

Graca Porto

Portugal

Milton Prabu

India

Jonato Prestes

Brazil

Resia Pretorius

South Africa

Gerald J. Prud'homme

Canada

Bolin Qin

United States of America

Jun Qin

United States of America

Ian Reid

New Zealand

Scott Reisman

United States of America

Patrick Rensen

Netherlands

Marisa Repetto

Argentina

Gerald Rimbach

Germany

Ulf Riserus

Sweden

Vladimir Ritov

United States of America

Roberto Rivabene

Italy

Lee Roberts

United Kingdom

Helen Roche

Ireland

Bernardo Rodriguez-Iturbe

Venezuela

Michael Rogers

Belgium

Suzanne Rogers

Australia

Yan Ronglin

China

Olav Rooyackers

Sweden

Jean-Max Rouanet

France

Iwona Rudkowska

Canada

Alan Ryan

United States of America

Craig Sale

United Kingdom

Honoo Satake

Japan

Edoardo Savarino

Italy

Adrienne Scheck

United States of America

Jochen Klaus Schubert

Germany

Matthew Schubert

Australia

Lai-Chu See

Taiwan

Akira Sekikawa

United States of America

Thomas Seyfried

United States of America

Abdul Rauf Shakoori

Pakistan

Ron Shaoul

Israel

Sushma Sharma

United States of America

Jayant Shenai

United States of America

Vanessa Sherk

United States of America

Yu Shi

United States of America

John L Sievenpiper

Canada

David Silver

Singapore

Adrian Slee

United Kingdom

David Smith

United Kingdom

Tiina Solakivi

Finland

YOONJU SONG

Korea South

Julia Spielmann

Germany

Rai Ajit Srivastava

United States of America

William Stanley

Australia

Rinke Stienstra

Netherlands

Jeff Stout

United States of America

Ryan Streeper

United States of America

Shi Sun

United States of America

Ivan Tancevski

Austria

Minghua Tang

United States of America

Motohiro Tani

Japan

Luc Tappy

Switzerland

Carla Taylor

Canada

Lemuel W Taylor

United States of America

Andrew Tee

United Kingdom

Daniel Teitelbaum

United States of America

Alexander Tenenbaum

Israel

Diana Thomas

United States of America

Jency Thomas

Australia

Michael Thorner

United States of America

Uwe Tietge

Netherlands

Marijana Todorcevic

United Kingdom

Ramin Tolouian

United States of America

Susan Torres

Australia

Janet Tou

United States of America

Donatella Tramontano

Italy

Angelo Tremblay

Canada

Antonia Trichopoulou

Greece

Antonio Valencia

United States of America

Angela Valverde

Spain

Klaske van Norren

Netherlands

Kristin Verbeke

Belgium

Victor Victor

Spain

Jeff Volek

United States of America

Andra Waagmeester

Netherlands

Yan Wang

United States of America

Craig Warden

United States of America

Kenchi Watanabe

Japan

Malcolm Watford

United States of America

Martin O. Weickert

United Kingdom

Peter JM Weijs

Netherlands

JoEllen Welsh

United States of America

Paul Williams

United States of America

David Wright

Canada

Wen-Huey Wu

Taiwan

Angela Wyse

Brazil

Guoliang Xia

China

Hariom Yadav

India

Kazumasa Yamagishi

United States of America

Jamey Young

United States of America

Michael Zemel

United States of America

Shuang-Qing Zhang

China

Xia Zhang

China

Yong Zhu

United States of America

